# Impact of body mass index on long-term outcomes in patients undergoing percutaneous coronary intervention stratified by diabetes mellitus: a retrospective cohort study

**DOI:** 10.1186/s12872-024-03770-w

**Published:** 2024-02-16

**Authors:** Chongyou Rao, Qin Zhong, Rilige Wu, Zongren Li, Yongjie Duan, You Zhou, Chi Wang, Xu Chen, Ruiqing Wang, Kunlun He

**Affiliations:** 1 Medical Big Data Research Center, Medical Innovation Research Division of Chinese, PLA General Hospital, 28 Fuxing RD, Beijing, 100853 China; 2 Graduate School of Chinese, PLA General Hospital, Beijing, 100853 China; 3 School of Medicine, Nankai University, Tianjin, 300071 China

**Keywords:** Body mass index, Diabetes mellitus, Obesity paradox, Percutaneous coronary intervention, Cardiovascular disease, Prognosis

## Abstract

**Background:**

Patients with diabetes mellitus (DM) caused by obesity have increased in recent years. The impact of obesity on long-term outcomes in patients undergoing percutaneous coronary intervention (PCI) with or without DM remains unclear.

**Methods:**

We retrospectively analysed data from 1918 patients who underwent PCI. Patients were categorized into four groups based on body mass index (BMI, normal weight: BMI < 25 kg/m^2^; overweight and obese: BMI ≥ 25 kg/m^2^) and DM status (presence or absence). The primary endpoint was the occurrence of major adverse cardiac and cerebrovascular events (MACCE; defined as all-cause death, myocardial infarction, stroke, and unplanned repeat revascularization).

**Results:**

During a median follow-up of 7.0 years, no significant differences in MACCE, myocardial infarction, or stroke were observed among the four groups. Overweight and obese individuals exhibited lower all-cause mortality rates compared with normal-weight patients (without DM: hazard ratio [HR]: 0.54, 95% confidence interval [CI]: 0.37 to 0.78; with DM: HR: 0.57, 95% CI: 0.38 to 0.86). In non-diabetic patients, the overweight and obese group demonstrated a higher risk of unplanned repeat revascularization than the normal-weight group (HR:1.23, 95% CI:1.03 to 1.46). After multivariable adjustment, overweight and obesity were not significantly associated with MACCE, all-cause death, myocardial infarction, stroke, or unplanned repeat revascularization in patients with and without diabetes undergoing PCI.

**Conclusion:**

Overweight and obesity did not demonstrate a significant protective effect on long-term outcomes in patients with and without diabetes undergoing PCI.

**Supplementary Information:**

The online version contains supplementary material available at 10.1186/s12872-024-03770-w.

## Introduction

Diabetes mellitus (DM) is closely related to a greater burden of atherosclerotic plaque and an increased risk of adverse clinical outcomes [1]. Over the past decades, the use of new-generation drug-eluting stents has resulted in substantial improvements in adverse cardiovascular outcomes among patients with post-percutaneous coronary intervention (PCI) [2]. Despite these advancements, the mortality risk and adverse cardiovascular outcomes after PCI remain significantly higher in patients with diabetes compared with their non-diabetic counterparts, even in the era of drug-eluting stents [3–5]. Furthermore, DM is frequently accompanied by several comorbidities and diverse clinical conditions, potentially complicating its clinical utility and interpretability within specific patient subgroups.

Obesity has emerged as a significant public health concern in China and is highly prevalent among patients undergoing PCI [6]. Existing research and multiple guidelines have demonstrated that obesity is an independent risk factor for coronary heart disease [7]. Additionally, prior investigations have unveiled a notable interaction between DM and obesity whereby their combined presence augments hypercoagulation, thereby increasing the incidence of adverse cardiovascular events [8]. Contrarily, an increasing number of clinical observational studies, including meta-analyses [9, 10], have reported that patients with obesity have better prognostic outcomes than those who are normal weight or underweight. This phenomenon is referred to as the "obesity paradox". Intriguingly, the contradictory obesity paradox phenomenon has also been observed in patients with diabetes undergoing PCI during long-term follow-up [11]. To date, most studies have primarily focused on independent risk factors within the PCI population, neglecting the potential interplay between DM and obesity and its ramifications for patients receiving PCI. The influence of concurrent DM and obesity on this phenomenon is yet to be thoroughly explored.

Given the rising global prevalence of DM and obesity [12], a growing proportion of patients undergoing PCI are anticipated to be affected by these conditions. Therefore, examining the implications of DM and obesity on clinical outcomes post-PCI is crucial for postoperative health management and the adjustment of treatment strategies in this patient population. In this study, we aimed to evaluate the influence of body mass index (BMI) on prognostic outcomes in patients post-PCI, stratified by DM, over an extended follow-up period (> 5 years). The findings of this research may contribute to a more nuanced understanding of these interactions, thereby informing the development of more personalized and effective treatment strategies.

## Methods

### Study design

This retrospective, observational study enrolled patients undergoing PCI at the Chinese PLA General Hospital (Beijing, China) between January 2007 and December 2014. Inclusion criteria comprised all patients aged 18 years or older who received drug-eluting stent implantation and who completed follow-up. Exclusion criteria were patients who simultaneously received metal or bioabsorbable stents, who had underlying malignancy and end-stage heart failure, and those without height or weight recorded. Underweight patients (BMI < 18.5 kg/m^2^) were excluded owing to the limited patient numbers (35 patients). Data extraction from medical records encompassed baseline characteristics, coronary lesion attributes, laboratory tests, procedural specifics, and discharge medications. BMI was calculated via the formula: weight (kg) divided by height (m) squared (kg/m^2^). According to World Health Organization (WHO) recommendations for managing obese populations [13], patients were classified into two categories: normal weight (BMI 18.5–24.9 kg/m^2^) and overweight and obese (BMI ≥ 25 kg/m^2^). The present study complied with the Strengthening the Reporting of Observational Studies in Epidemiology (STROBE) guidelines [14]. The Chinese PLA General Hospital's Clinical Research Ethics Committee approved this study (S2017-035–01). Because of the retrospective design of this study, the need for informed consent was waived by the institutional review board, and information related to patient identity was concealed.

### Definitions and clinical endpoints

DM was defined as a history of DM or the current use of anti-diabetes therapy [15]. Hypertension was identified as a history of hypertension and/or current receiving anti-hypertensive treatment [16]. Dyslipidaemia was defined as total cholesterol ≥ 6.2 mmol/L (240 mg/dL) and/or triglycerides ≥ 2.3 mmol/L (200 mg/dL) and/or high-density lipoprotein cholesterol (HDL-C) < 1.0 mmol/L (40 mg/dL) and/or low-density lipoprotein cholesterol ≥ 4.1 mmol/L (160 mg/dL) [17] and/or treatment with dyslipidemia medication. An estimated glomerular filtration rate < 60 mL/min/1.73 m^2^ was used to define renal insufficiency [18]. We used the Charlson Comorbidity Index to assess comorbidity [19]. Smoking was defined as current smoking or cessation for less than 6 months. Multi-vessel coronary disease referred to coronary stenoses ≥ 50% in two or more of the three major epicardial arteries or their major side branches measuring ≥ 2.0 mm in diameter [20].

The primary endpoint was major adverse cardiac and cerebrovascular events (MACCE), defined as all-cause death, myocardial infarction (MI), stroke, and unplanned repeat revascularization (URR). The secondary endpoint comprised each component of the primary outcome. MI was classified as either ST-segment elevation myocardial infarction (STEMI) or non-ST-segment elevation myocardial infarction (NSTEMI), according to the Fourth Universal Definition of Myocardial Infarction [21]. Stroke was determined as ischemic stroke via imaging, confirmed by a neurologist. URR encompassed any non-staged ischemia-driven coronary revascularization post-PCI (percutaneous or surgical). Patient follow-up was executed via telephone contact and medical record examination and was independently conducted by trained researchers blinded to patient information. All follow-up data underwent independent assessment by at least two researchers to ensure quality control for data entry and variable consistency checks.

### Statistical analysis

Continuous variables are expressed as mean and standard deviation and categorical variables are presented as percentages. Group differences were compared using Mann–Whitney U or *t*-tests for continuous variables and Pearson's χ^2^ or Fisher's exact tests for categorical variables. The primary and secondary outcomes were stratified by BMI groups for all analyses. Long-term outcomes of interest are reported as Kaplan–Meier estimates and log-rank test. A Cox proportional hazards model was used to estimate the hazard ratio (HR) of outcomes among groups. If the same event occurred more than twice, the first event was used in the analysis. Adjusted models were adjusted for age, sex, smoking, previous MI, hypertension, cerebrovascular disease, peripheral vascular disease, renal insufficiency, albumin, cardiac ejection fraction, multi-vessel coronary disease, and primary PCI. The proportional hazards assumption was assessed using Schoenfeld residual plots and the Grambsch and Therneau test. To handle missing values, multiple imputations were implemented using random forests with the 'mice' package in R. The algorithm's performance was assessed through convergence diagnostics and the distribution of imputed variables. Imputation was conducted using 20 imputed datasets, and results were combined using Rubin's rules to account for uncertainty introduced by the imputation process. RStudio 4.1.3 (The R Project for Statistical Computing) was used for the statistical analysis, and a two-sided *P*-value < 0.05 was considered statistically significant.

## Results

### Study cohort

**Fig. 1:**
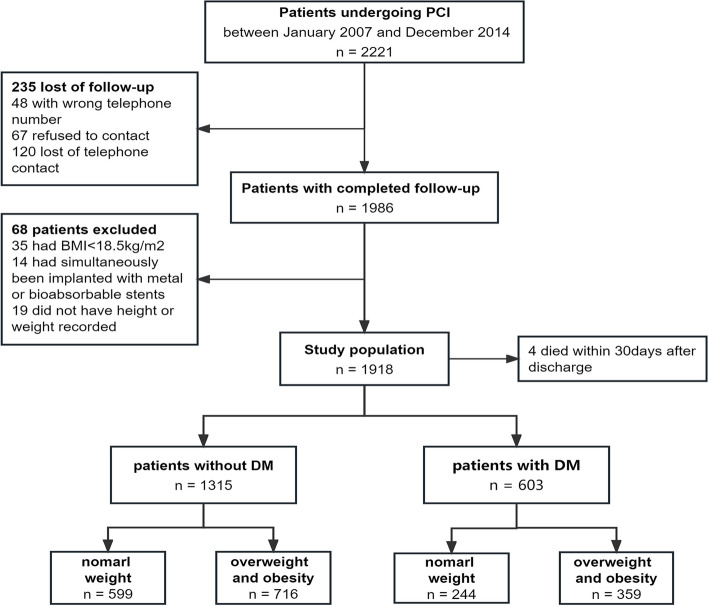
Study Flowchart. PCI, percutaneous coronary intervention; BMI, body mass index; DM, diabetes mellitus

A total of 1918 patients who underwent PCI between January 2007 and December 2014 were included in this study (Fig. 1). The median follow-up time was 7 years (interquartile range 5.68–8.01 years). Among these patients, 603 (31.4%, 62 with previously unrecognized DM) had DM, and 1315 (68.6%) patients did not have DM. Tables 1 and 2 display the baseline characteristics of patients stratified by DM and BMI groups. In patients with and without diabetes, individuals with overweight and obesity (BMI ≥ 25 kg/m^2^) were consistently characterized by younger age, a higher proportion of male individuals, higher albumin and triglyceride levels, and lower HDL-C levels, compared with those who had normal weight (BMI 18.5–24.9 kg/m^2^, *P* < 0.05, Table 1).

**Table 1:** Baseline clinical characteristics by BMI and DM status

Variables		**DM**(*n* = 603)			**Non-DM**(*n* = 1315)		
	**Overall** (*n* = 1918)	**Normal-weight** (*n* = 244)	**Overweight and Obesity** (*n* = 359)	***P*** value	**Normal-weight** (*n* = 599)	**Overweight** **and Obesity** (*n* = 716)	***P*** value
Age, y	60.3(11.3)	65.0(10.0)	60.7(10.9)	** < 0.001**	61.3(11.1)	57.5(11.5)	** < 0.001**
Men, No. (%)	1462 (76.2)	151 (61.9)	264 (73.5)	**0.003**	459 (76.6)	588(82.1)	**0.017**
Clinical presentation, No. (%)							
CCS	142 (7.4)	18 (7.4)	34 (9.5)	0.453	35 (5.8)	55 (7.7)	0.228
UA	869 (45.3)	113 (46.3)	165 (46.0)	0.999	262 (43.7)	329(45.9)	0.455
STEMI	644 (33.6)	77 (31.6)	104 (29.0)	0.555	215 (35.9)	248(34.6)	0.677
NSTEMI	263 (13.7)	36 (14.8)	56 (15.6)	0.867	87 (14.5)	84 (11.7)	0.156
Concomitant disease, No. (%)							
Hypertension	1047 (54.6)	138 (56.6)	222 (61.8)	0.225	287 (47.9)	400(55.9)	**0.005**
Hyperlipidemia	381 (19.9)	46 (18.9)	68 (18.9)	1	124 (20.7)	143(20.0)	0.796
Stroke	242 (12.6)	32 (13.1)	56 (15.6)	0.465	70 (11.7)	84 (11.7)	1
RI	152(7.9)	32(13.1)	38(10.6)	0.411	46(7.7)	36(5.0)	0.062
PVD	111 (5.8)	24 (9.8)	17 (4.7)	**0.023**	38 (6.3)	32 (4.5)	0.166
CCI	1.36 (1.05)	2.16 (1.03)	2.07 (0.96)	0.273	1.07 (0.91)	0.98(0.87)	0.088
Smoking status, No. (%)							
Never	1104 (57.6)	162 (66.4)	222 (61.8)	0.521	354 (59.1)	366(51.1)	**0.001**
Former smoker	352 (18.4)	41(16.8)	68 (18.9)		114 (19.0)	129(18.0)	
Current smoker	462 (24.1)	41(16.8)	69 (19.2)		131 (21.9)	221(30.9)	
Clinical history, No. (%)							
Previous MI	149 (7.8)	16 (6.6)	33 (9.2)	0.312	46 (7.7)	54 (7.5)	1
Previous PCI	87 (4.5)	10 (4.1)	24 (6.7)	0.241	30 (5.0)	23 (3.2)	0.131
Previous CABG	23 (1.2)	1 (0.4)	3 (0.8)	0.904	7 (1.2)	12 (1.7)	0.592
Laboratory and Echocardiogram							
ALT, IU/L	32.76(29.47)	29.91(29.83)	29.74(25.40)	0.939	31.76(32.20)	36.28(28.79)	**0.007**
ALB, g/L	40.82(3.80)	40.06 (4.25)	40.93(3.88)	**0.01**	40.44(3.87)	41.38(3.49)	** < 0.001**
Cr, mmol/L	81.32(54.69)	84.67(73.67)	84.26(68.04)	0.944	79.45(44.49)	80.29(46.84)	0.739
TG, mmol/L	1.61 (0.94)	1.62 (0.90)	1.80 (1.13)	**0.038**	1.39 (0.74)	1.70(0.97)	** < 0.001**
TC, mmol/L	4.27 (1.06)	4.28 (1.10)	4.21 (1.06)	0.466	4.21 (1.02)	4.28(1.05)	0.237
HDL-C, mmol/L	1.05 (0.27)	1.06 (0.27)	0.99 (0.24)	**0.001**	1.10 (0.29)	1.02(0.26)	** < 0.001**
LDL-C, mmol/L	2.59 (0.89)	2.54 (0.92)	2.54 (0.88)	0.965	2.55 (0.88)	2.61(0.84)	0.248
EF, %	55.72(8.53)	55.36 (8.91)	55.06(8.70)	0.683	55.38(8.89)	56.35(8.06)	**0.039**
Discharge prescription, No. (%)							
Aspirin	1910 (99.6)	243 (99.6)	358 (99.7)	1	595 (99.3)	714 (99.7)	0.529
Clopidogrel	1878 (97.9)	242 (99.2)	353 (98.3)	0.593	587 (98.0)	696 (97.2)	0.456
Ticagrelor	39 (2.0)	2 (0.8)	6 (1.7)	0.593	11 (1.8)	20 (2.8)	0.339
Statin	1896 (98.9)	243 (99.6)	354 (98.6)	0.438	588 (98.2)	711 (99.3)	0.105
Beta-blocker	1526 (79.6)	192 (78.7)	285 (79.4)	0.916	467 (78.0)	582 (81.3)	0.154
ACEI/ARB	1077 (56.2)	142 (58.2)	231 (64.3)	0.15	306 (51.1)	398 (55.6)	0.115
Oral anti-diabetic drugs	428 (71.0)	162 (66.4)	266 (74.1)	0.051	-	-	-
Insulin therapy	264 (43.8)	121 (49.6)	143 (39.8)	**0.022**	-	-	-

*DM* diabetes mellitus, *BMI* body mass index, *CCS* chronic coronary syndrome, *UA* unstable angina, *STEMI* ST-segment elevation myocardial infarction, *NSTEMI* non-ST-segment elevation myocardial infarction, *RI* renal insufficiency, *PVD* peripheral vascular disease, *CCI* Charlson Comorbidity Index, *MI* myocardial infarction, *PCI* percutaneous coronary intervention, *CABG* coronary artery bypass grafting, *ALT* alanine transferase, *ALB* albumin, *Cr* creatinine, *TG* triglycerides, *TC* total cholesterol, *HDL-C* high-density lipoprotein-cholesterol, *LDL-C* low-density lipoprotein-cholesterol, *EF* ejection fraction, *ACEI/ARBs* angiotensin-converting enzyme inhibitors and angiotensin receptor blockages

Bold indicates *P* < 0.05

**Table 2:** Angiographic and procedural characteristics by BMI and DM status

Variables		**DM**			**Non-DM**		
	**Overall** (*n* = 1918)	**Normal-** **weight** (*n* = 244)	**Overweight and Obesity** (*n* = 359)	***P*** value	**Normal-** **weight** (*n* = 599)	**Overweight and Obesity** (*n* = 716)	***P*** value
Lesion Characteristics, No. (%)							
Single-vessel	470 (24.5)	31 (12.7)	72 (20.1)	**0.025**	170 (28.4)	197 (27.5)	0.774
Multi-vessel	1443(75.2)	213 (87.3)	286 (79.7)	**0.02**	425 (71.0)	519 (72.5)	0.579
Double-vessel	583 (30.4)	71 (29.1)	96 (26.7)	0.588	200 (33.4)	216 (30.2)	0.233
Triple-vessel	860 (44.8)	142 (58.2)	190 (52.9)	0.232	225 (37.6)	303 (42.3)	0.09
LM	167 (8.7)	30 (12.3)	30 (8.4)	0.148	53 (8.8)	54 (7.5)	0.446
Total stent length, mm	51.73(34.47)	59.11(36.46)	54.74(37.68)	0.158	47.20(29.87)	51.50(35.17)	**0.018**
Minimum stent diameter, mm	2.87 (0.43)	2.74 (0.35)	2.83 (0.42)	**0.012**	2.91 (0.43)	2.91 (0.45)	0.763
Stents implanted, No. (%)	2.09 (1.23)	2.36 (1.27)	2.20 (1.34)	0.143	1.90 (1.06)	2.09 (1.27)	**0.005**
Stent type implanted							
Early-generation	151 (7.9)	12 (4.9)	21 (5.8)	0.756	52 (8.7)	66 (9.2)	0.809
New-generation	1676 (87.4)	224 (91.8)	322 (89.7)	0.467	525 (87.6)	605 (84.5)	0.12

*BMI* body mass index, *DM* diabetes mellitus, *LM* left main disease

Bold indicates *P* < 0.05

More than 90% of patients were admitted with acute coronary syndrome, with no difference in the distribution of STEMI, NSTEMI, and unstable angina among the four groups. In the subgroup of patients with diabetes, individuals with overweight and obesity were less likely to receive insulin therapy and to have peripheral vascular disease, multi-vessel coronary disease than normal-weight individuals (*P* < 0.05). The minimum stent diameter was larger in individuals with overweight and obesity than in those with normal weight. However, in the subgroup of patients without DM, overweight and obese individuals were more likely to have hypertension and a higher level of alanine transferase and ejection fraction (*P* < 0.05). The number and length of stents were greater and longer, respectively, in the overweight and obesity group than in the normal-weight group (*P* < 0.05). There was no significant difference in discharge prescription between the two BMI categories according to different DM states (Table 2).

Supplementary Tables 1 and 2 summarize the baseline data, severity of disease, and procedural characteristics by BMI category. Patients with BMI ≥ 25 kg/m^2^ were younger (age 58.6 ± 11.4 vs. 62.4 ± 10.9 years; *P* < 0.001), more likely to be male individuals (79.3% vs. 72.4%; *P* = 0.001), and more likely to have cardiovascular risk factors such as smoking, hypertension, and DM (*P* < 0.05), compared with normal-weight patients. However, patients with normal weight were more likely to have peripheral vascular disease than overweight and obese patients (4.6% vs. 7.4%; *P* < 0.05). Patients with BMI ≥ 25 kg/m^2^ exhibited higher levels of triglycerides, alanine transferase, and albumin (*P* < 0.05). In contrast, HDL-C levels were higher in normal-weight patients (1.10 ± 0.29 mmol/L vs. 1.02 ± 0.25 mmol/L; *P* < 0.001). No significant differences in the use of other therapeutic medications were found between the two groups, except for greater use of oral anti-diabetes drugs and angiotensin-converting enzyme inhibitors/angiotensin receptor blockages at discharge in patients with BMI ≥ 25 kg/m^2^. Lesion characteristics and stent details, such as a single-vessel lesion, multi-vessel lesion, left main coronary lesion, total stent length, minimum stent diameter, number of implanted stents, and implanted stent type showed no significant differences between the two groups by BMI category (Supplementary Table 2).

### Clinical outcomes

**Fig. 2:**
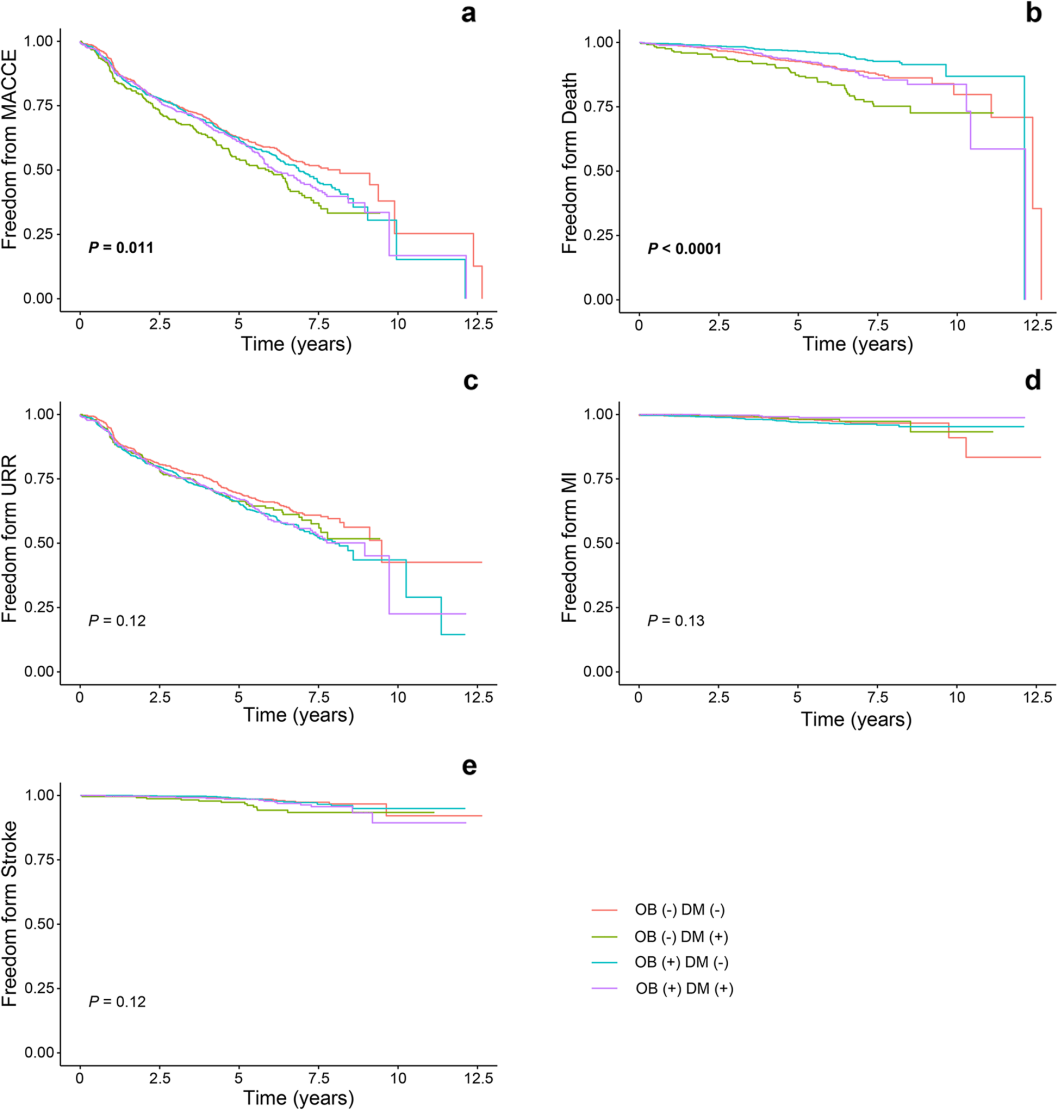
Cumulative ratio of freedom from clinical outcomes among the 4 groups. **a**, Cumulative ratio of freedom from MACCE among the 4 groups (*P* = 0.011). **b**, Cumulative ratio of freedom from all-cause death among the 4 groups (*P* < 0.0001). **c**, Cumulative ratio of freedom from URR among the 4 groups (*P* = 0.12). **d**, Cumulative ratio of freedom from MI among the 4 groups (*P* = 0.13). **e**, Cumulative ratio of freedom from stroke among the 4 groups (*P* = 0.12). MACCE, major adverse cardiac and cerebrovascular events; MI, myocardial infarction; URR, unplanned repeat revascularization; OB, obese; DM, diabetes mellitus

Table 3 shows crude rates of the clinical outcomes at a median follow-up of 7.0 years, according to BMI. All-cause mortality was higher in patients with normal weight compared with those in the overweight and obesity group (14.2% vs. 8.5%; *P* < 0.001). However, compared with the normal-weight group, the URR rate was higher in the overweight and obesity group (35.8% vs. 42.3%; *P* = 0.004). Rates of MACCE, MI, and stroke did not differ significantly between patients with normal weight and those with overweight and obesity. Figure 2 shows the cumulative rate of freedom from MACCE, all-cause death, URR, MI, and stroke among the four groups. Freedom from MACCE and all-cause death were lowest in the DM alone group (*P* = 0.011, *P* < 0.0001, respectively); however, no differences were found in URR, MI, and stroke rate among the four groups (*P* = 0.12, *P* = 0.13 and *P* = 0.12*,* respectively). Table 4 shows the incidence of adverse clinical outcomes and unadjusted and adjusted HR in patients with and without DM, according to BMI. Before the multivariate correction, in patients with diabetes, the overweight and obesity group was associated with lower all-cause mortality compared with the normal-weight group (HR: 0.57, 95% confidence interval [CI]: 0.38 to 0.86]). Similarly, in non-diabetic patients, the overweight and obesity group was associated with lower all-cause mortality compared with the normal-weight group (HR: 0.54, 95% CI: 0.37 to 0.78). Additionally, overweight and obesity were significantly associated with the risk of future URR only in patients with diabetes (HR: 1.23, 95% CI: 1.03 to 1.46). However, these associations were not significant after multivariate adjustment (*P* > 0.05). Furthermore, overweight and obesity were not significantly associated with MACCE, MI, and stroke in patients with and without diabetes who were undergoing PCI either before or after multi-variate adjustment (*P* > 0.05). Overall, the effect of obesity on both primary and secondary outcome events did not differ significantly between different diabetes states (*P*-interaction > 0.1).

**Table 3:** Outcomes analyses according to BMI

Outcomes	BMI Category	Event Rate n(%)	***P *value**
MACCE	Normal-weight	410 (48.6%)	0.418
	Overweight and Obesity	544 (50.6%)	
All-cause death	Normal-weight	120 (14.2%)	** < 0.001**
	Overweight and Obesity	91 (8.5%)	
MI	Normal-weight	24 (2.8%)	1
	Overweight and Obesity	30 (2.8%)	
Stroke	Normal-weight	26 (3.1%)	0.998
	Overweight and Obesity	32 (3.0%)	
URR	Normal-weight	302 (35.8%)	**0.004**
	Overweight and Obesity	455 (42.3%)	

*BMI* body Mass Index, *MACCE* major adverse cardiac and cerebrovascular events, *MI* myocardial infarction, *URR* unplanned repeat revascularization

Bold indicates *P* < 0.05

**Table 4:** Outcomes analyses according to BMI and DM status

**Outcomes**	**DM Category**	**BMI Category**	**Event Rate** n (%)	**Unadjusted Model**			**Adjusted Model**			***P-***interaction
				HR	95% CI	***P ***value	HR	95% CI	***P ***value	
MACCE	Non-DM	Normal-weight	272 (45.4%)	1(Reference)						0.158
		Overweight and Obesity	351 (49.0%)	1.12	0.96, 1.32	0.15	1.10	0.94, 1.30	0.2	
	DM	Normal-weight	138 (56.5%)	1(Reference)						
		Overweight and Obesity	193 (53.7%)	0.86	0.69, 1.07	0.2	0.93	0.74, 1.18	0.6	
All-cause death	Non-DM	Normal-weight	71 (11.8%)	1(Reference)						0.833
		Overweight and Obesity	45 (6.2%)	0.54	0.37, 0.78	**0.001**	0.71	0.48, 1.04	0.078	
	DM	Normal-weight	49 (20.0%)	1(Reference)						
		Overweight and Obesity	46 (12.8%)	0.57	0.38, 0.86	**0.007**	0.75	0.49, 1.16	0.2	
MI	Non-DM	Normal-weight	18 (3.0%)	1(Reference)						0.126
		Overweight and Obesity	26 (3.6%)	1.19	0.65, 2.16	0.6	1.16	0.62, 2.16	0.6	
	DM	Normal-weight	6 (2.4%)	1(Reference)						
		Overweight and Obesity	4 (1.1%)	0.42	0.12, 1.48	0.2	0.37	0.10, 1.38	0.14	
Stroke	Non-DM	Normal-weight	14 (2.3%)	1(Reference)						0.361
		Overweight and Obesity	19 (2.6%)	1.09	0.55, 2.18	0.8	1.31	0.63, 2.70	0.5	
	DM	Normal-weight	12 (4.9%)	1(Reference)						
		Overweight and Obesity	13 (3.6%)	0.65	0.30, 1.43	0.3	1.14	0.95, 1.36	0.20	
URR	Non-DM	Normal-weight	212 (35.3%)	1(Reference)						0.545
		Overweight and Obesity	304 (42.4%)	1.23	1.03, 1.46	**0.022**	1.06	0.81, 1.40	0.70	
	DM	Normal-weight	90 (36.8%)	1(Reference)						
		Overweight and Obesity	151 (42.0%)	1.06	0.81, 1.37	0.7	1.11	0.84, 1.48	0.5	

Covariates for the adjusted model: age, sex, smoking, previous myocardial infarction, hypertension, cerebrovascular disease, peripheral vascular disease, renal insufficiency, albumin, cardiac ejection fraction, multi-vessel coronary disease, and primary percutaneous coronary intervention

*BMI* body mass index, *DM* diabetes mellitus, *CI* confidence interval, *HR* hazard ratio, *MACCE* major adverse cardiac and cerebrovascular events, *MI* myocardial infarction, URR unplanned repeat revascularization

Bold indicates *P* < 0.05

Supplementary Table 3 listed covariates for the adjusted model. Based on the results from the Cox proportional hazard model, we found that hypertension, renal insufficiency, and multi-vessel coronary disease were predictors of MACCE (HR: 1.25, 1.34, and 1.27, respectively), and albumin was associated with reduced risk of MACCE (HR: 0.98). Age, previous MI, peripheral vascular disease history, renal insufficiency history, and multi-vessel coronary disease were predictors of all-cause death (HR: 1.06, 1.61, 1.68, 2.68, and 1.52, respectively), Albumin and cardiac ejection fraction could reduce the risk of all-cause death (HR: 0.95 and 0.98, respectively). Moreover, albumin could also reduce the risk of MI (HR:0.86). Age and cerebrovascular disease history were predictors of stroke. Finally, hypertension, multi-vessel coronary disease, and cardiac ejection fraction were associated with unplanned repeat revascularization (HR:1.25, 1.26, and 1.01, respectively). Additionally, the present study included 97 patients who had acute heart failure during their hospitalization, as well as 8 patients who had chronic heart failure with reduced ejection fraction in long-term follow-up. A sensitivity analysis was performed after removing these patients, and the results were consistent with the previous findings. Supplementary Table 4 provides more information on the results.

## Discussion

In this retrospective investigation, we analysed long-term clinical outcomes in patients who underwent PCI, aiming to discern the prognostic implications of DM in relation to BMI in the era of contemporary drug-eluting stents. Our primary finding was that after a median follow-up of 7 years, our study did not demonstrate a significant "protective effect" of overweight and obesity in patients undergoing PCI, irrespective of their DM status.

Patients with DM undergoing PCI consistently exhibit a higher incidence of adverse clinical outcomes [5]. Reduced microvascular perfusion owing to endothelial dysfunction and reduced blood flow reserve may lead to a poor response to reperfusion therapy and an increased risk of cardiac pump failure and death in patients with diabetes [22, 23]. Furthermore, these patients are more likely to have complex coronary artery lesions, such as multi-vessel and diffuse coronary lesions [24]. The increased risk of repeated revascularization owing to complex coronary lesions raises the risk of cardiovascular death four-fold [25].

Obesity is an established risk factor for DM, with obese individuals having a six-fold higher overall risk of DM than those of normal weight [26]. Obesity may also cause a continued increase in platelet reactivity in patients with diabetes. Insulin resistance associated with obesity can increase platelet reactivity, resulting in a lack of aspirin efficacy [27], which may increase the risk of subsequent cardiac events in patients with type 2 DM. However, this is inconsistent with the results of a recent large-sample clinical observational study [11].

Prior studies have not extensively explored the potential interactions between DM and obesity and their collective impact on patients undergoing PCI. Terada and colleagues [11] reported contradictory findings in their study investigating the impact of DM and obesity status on adverse clinical outcomes in patients with coronary revascularization. Those authors found that overweight patients had better long-term survival in a subgroup of 6793 patients undergoing PCI, even after correcting for other risk factors (5 years: HR 0.80, 95% CI: 0.66–0.99; 10 years: HR 0.82, 95% CI: 0.68–0.99). However, the authors did not adjust for multi-vessel coronary disease, which could increase the risk of adverse clinical outcomes [28]. The discrepancy with our results may be attributed to the Asian-majority population in our study, where the obesity paradox is not typically observed owing to low rates of extreme obesity [29]. It is generally recognized that obese patients are less active and have a poorer exercise tolerance than those with normal body weight, however, obese patients with younger age are more likely to present cardiorespiratory fitness, which may change the relationship between obesity and outcomes. Additionally, normal-weight patients co-exist with unobserved confounding variables such as frailty or differences in genetic susceptibility may also lead to inconsistent results.

The obesity paradox phenomenon reported in previous studies [9–11, 30] may reflect limitations inherent in the analysis of clinical observational studies, such as confounding bias and collider stratification bias. Although some authors hypothesize that potential pathophysiological mechanisms support the biological plausibility of the obesity paradox, others remain skeptical and provide methodological explanations [7, 10]. Confounding factors in the obesity–death relationship include age, sex, race and ethnicity, smoking status, alcohol consumption, income, education, physical activity, and dietary patterns [31]. Uncontrolled confounding variables could lead to bias [26]. Additionally, reverse causality may cause bias where a pre-existing disease causes unexpected weight loss and higher mortality, making obesity appear protective [1, 8].

In previous studies, certain trends have been observed: in comparison with their obese counterparts, normal-weight patients are typically older [10, 11, 31] and have a higher incidence of renal insufficiency, peripheral vascular disease, and major bleeding events [11, 32, 33]. Conversely, overweight or obese patients have a lower risk of cardiogenic shock, post-hospital cardiac arrest [32, 34], smaller infarct size after myocardial infarction [35], more aggressive use of invasive surgery [36], and more favorable secondary prevention treatment [32]. Given the numerous differences between obese and non-obese patients, statistical adjustment for risk factors is necessary. In our study, after adjusting for major comorbidities, smoking, and the number of narrow coronary vessels, the "obesity paradox" of overweight or obesity was no longer significant. Therefore, this phenomenon may reflect the baseline difference in risk profiles.

The number of patients with DM caused by obesity has increased at an unprecedented rate in recent years [37], and the association between morbid obesity (BMI ≥ 40.0 kg/m^2^) and adverse cardiovascular outcomes has been identified. Previous studies have demonstrated higher long-term mortality in patients with extreme obesity after PCI [11, 38]. However, the impact of overweight and mild-to-moderate obesity on cardiovascular events in patients undergoing PCI remains controversial. Regardless of the authenticity of the "obesity paradox", the evidence supporting this phenomenon should not be misinterpreted as advocating high BMI targets in patients undergoing PCI. Instead, future studies need to investigate why patients with higher BMI seem to have a lower risk of cardiovascular events than those with normal BMI and to identify confounding factors with potential protective or pathogenic effects in overweight and obese patients. Although the present study did not identify a "protective effect" of overweight and obesity, our study and previous studies [11] have not found an increased risk of adverse clinical outcomes in overweight patients with diabetes undergoing PCI. The question of whether overweight and obesity a protective or risk factors remains unanswered. To fully elucidate the prognostic effect of DM and obesity status in patients with PCI, more extensive prospective trials are needed to fully elucidate the mechanisms that underlie the impact of DM and obesity on patients undergoing PCI and to avoid potential bias inherent in previous studies.

## Study limitations

Our findings warrant careful interpretation in light of several notable limitations. First, inherent to any retrospective study, potential confounding factors could influence the outcomes of the present investigation. Although multivariate Cox regression was performed to minimize the impact of potentially confounding variables, unmeasured variables still need to be clarified in the results. Future investigations should consider larger sample sizes and multi-center studies. Second, our study relied on BMI classifications derived from WHO recommendations to categorize patients' obesity status. However, alternative classification indices, such as waist circumference, body roundness index, and body size index, may also offer valuable insights into the pathophysiological distinctions associated with obesity. We recommend incorporating additional obesity indicators in subsequent studies to enhance the robustness of the findings [39]. Third, the BMI measurements and DM diagnosis were obtained only at baseline, the new onset DM and changes in BMI resulting from lifestyle changes during follow-up and their association with clinical outcomes warrant investigation in the future. Fourth, since patients with BMI > 25 kg/m^2^ were younger (age 58.6 vs 62.4, *P* < 0.001), the possibility of leading time bias for all-cause death and stroke cannot be excluded in the current study. In addition, we used specific criteria for the Chinese population rather than the current National Cholesterol Education Program (NCEP) Adult Treatment Panel (ATP) III guidelines to define dyslipidemia. Data entry bias might exist, and the results might be confounded if dyslipidemia was redefined. Last, a potential selection bias could be caused by missing data on height or weight. However, the missing proportion was only 0.1%, and nine people were excluded from the final analysis. No significant differences were found after performing a sensitivity analysis. Therefore, the deletion was considered within a tolerable range.

## Conclusion

Overweight or obesity did not exhibit a significant protective effect on long-term clinical outcomes in patients with and without diabetes who were undergoing PCI. Until further information is available, aggressive lifestyle modification and standard secondary prevention for coronary heart disease to reduce the risk of adverse clinical outcomes after PCI remain necessary in overweight or obese patients with DM.

### Supplementary Information

**Additional file 1:** **Supplementary Table 1.** Baseline clinical characteristics by BMI. **Supplementary Table 2.** Angiographic and Procedural Characteristics by BMI. **Supplementary Table 3.** Significant covariates in a Cox proportion hazard model for clinical outcomes. **Supplementary Table 4.** Sensitivity analysis for deleting heart failure patients.

## Data Availability

The data used in this study can be obtained from the corresponding author upon reasonable request.

## Abbreviations

BMI: Body mass index
CI: Confidence interval
DM: Diabetes mellitus
HDL-C: High-density lipoprotein cholesterol
HR: Hazard ratio
MACCE: Major adverse cardiac and cerebrovascular events
MI: Myocardial infarction
NSTEMI: Non-ST-segment elevation myocardial infarction
PCI: Percutaneous coronary intervention
STEMI: ST-segment elevation myocardial infarction
URR: Unplanned repeat revascularization
WHO: World Health Organization
