# Supplementary material for: Impact of body mass index on long-term outcomes in patients undergoing percutaneous coronary intervention stratified by diabetes mellitus: a retrospective cohort study
Source: BMC Cardiovasc Disord. 2024 Feb 16;24:113. doi: 10.1186/s12872-024-03770-w (PMC10874050; doi:10.1186/s12872-024-03770-w)
Supplement: Supplementary file 1 — Additional file 1: Supplementary Table 1. Baseline clinical characteristics by BMI. Supplementary Table 2. Angiographic and Procedural Characteristics by BMI. Supplementary Table 3. Significant covariates in a Cox proportion hazard model for clinical outcomes. Supplementary Table 4. Sensitivity analysis for deleting heart failure patients. [file 12872_2024_3770_MOESM1_ESM.docx]

| Supplementary Table 1. Baseline clinical characteristics by BMI | | | |
| --- | --- | --- | --- |
| Variables | **Normal-weight**  (n=843) | **Overweight and Obesity**  (n=1075) | ***P***  value |
| Age, y | 62.4 (10.9) | 58.6(11.4) | **<0.001** |
| Male, No. (%) | 610 (72.4) | 852 (79.3) | **0.001** |
| Clinical presentation, No. (%) | | | |
| CCS | 53 (6.3) | 89 (8.3) | 0.117 |
| UA | 375 (44.5) | 494 (46.0) | 0.552 |
| STEMI | 292 (34.6) | 352 (32.7) | 0.41 |
| NSTEMI | 123 (14.6) | 140 (13.0) | 0.356 |
| Concomitant disease, No. (%) | | | |
| Hypertension | 425 (50.4) | 622 (57.9) | **0.001** |
| DM | 244 (28.9) | 359 (33.4) | **0.042** |
| Hyperlipidemia | 170 (20.2) | 211 (19.6) | 0.814 |
| Stroke | 102 (12.1) | 140 (13.0) | 0.592 |
| RI | 78(9.3) | 74(6.9) | 0.069 |
| PVD | 62 (7.4) | 49 (4.6) | **0.012** |
| CCI | 1.38 (1.07) | 1.35 (1.04) | 0.439 |
| Smoking status, No. (%) | | | |
| Never | 516 (61.2) | 588 (54.7) | **0.003** |
| Former smoking | 155 (18.4) | 197 (18.3) |  |
| Current smoking | 172 (20.4) | 290 (27.0) |  |
| Clinical history, No. (%) | | | |
| Previous MI | 62 (7.4) | 87 (8.1) | 0.608 |
| Previous PCI | 40 (4.7) | 47 (4.4) | 0.78 |
| Previous CABG | 8 (0.9) | 15 (1.4) | 0.496 |
| Laboratory and Echocardiogram | | | |
| ALT, IU/L | 31.09 (31.38) | 34.07 (27.82) | **0.028** |
| ALB, g/L | 40.32 (3.95) | 41.21 (3.62) | **<0.001** |
| Cr, mmol/L | 80.85 (54.57) | 81.69 (54.81) | 0.739 |
| TG, mmol/L | 1.47 (0.81) | 1.72 (1.02) | **<0.001** |
| TC, mmol/L | 4.24 (1.04) | 4.29 (1.08) | 0.362 |
| HDL-C, mmol/L | 1.10 (0.29) | 1.02 (0.25) | **<0.001** |
| LDL-C, mmol/L | 2.56 (0.88) | 2.61 (0.89) | 0.294 |
| EF, % | 55.51 (8.86) | 55.88 (8.26) | 0.356 |
| Discharge prescription, No. (%) | | | |
| Aspirin | 838 (99.4) | 1072 (99.7) | 0.482 |
| Clopidogrel | 829 (98.3) | 1049 (97.6) | 0.321 |
| Ticagrelor | 13 (1.5) | 26 (2.4) | 0.235 |
| Statin | 831 (98.6) | 1065 (99.1) | 0.429 |
| Beta-blocker | 659 (78.2) | 867 (80.7) | 0.201 |
| ACEI/ARBs | 448 (53.1) | 629 (58.5) | **0.021** |
| Oral anti-diabetic drugs | 162 (19.2) | 266 (24.7) | **0.005** |
| Insulin therapy | 121 (14.4) | 143 (13.3) | 0.551 |
| BMI, body mass index; CCS, chronic coronary syndrome; UA, unstable angina; STEMI, ST-segment elevation myocardial infarction; NSTEMI, non-ST-segment elevation myocardial infarction; DM, diabetes mellitus; RI, renal insufficiency; PVD, peripheral vascular disease; CCI, Charlson Comorbidity Index; MI, myocardial infarction; PCI, percutaneous coronary intervention; CABG, coronary artery bypass grafting; ALT, alanine transferase; ALB, albumin; Cr, creatinine; TG, triglycerides; TC, total cholesterol; HDL-C, high-density lipoprotein- cholesterol; LDL-C, low-density lipoprotein-cholesterol; EF, ejection fraction; ACEI/ARBs, angiotensin-converting enzyme inhibitors and angiotensin receptor blockages; Bold indicates *P* < 0.05. | | | |

| Supplementary Table 2. Angiographic and Procedural Characteristics by BMI | | | |
| --- | --- | --- | --- |
| **Variables** | **Normal-weight**  (n=843) | **Overweight and Obesity**  (n=1075) | ***P***  value |
| Lesion Characteristics, No. (%) | | | |
| Single-vessel | 201 (23.8) | 269 (25.0) | 0.587 |
| Multi-vessel | 638 (75.7) | 805 (74.9) | 0.727 |
| Left main coronary | 83 (9.8) | 84 (7.8) | 0.138 |
| Stent Details | | | |
| Total stent length, mm | 50.65 (32.35) | 52.58 (36.04) | 0.222 |
| Minimum stent diameter, mm | 2.86 (0.41) | 2.88 (0.44) | 0.448 |
| Stents implanted, No. (%) | 2.04 (1.14) | 2.12 (1.29) | 0.115 |
| Stent type implanted | | | |
| Early generation DES | 64 (7.6) | 87 (8.1) | 0.75 |
| New-generation DES | 749 (88.8) | 927 (86.2) | 0.1 |
| BMI, body mass index; DES, drug-eluting sent | | | |

| Supplementary Table 3. Significant covariates in a Cox proportion hazard model for clinical outcomes | | | | | | | | | | |
| --- | --- | --- | --- | --- | --- | --- | --- | --- | --- | --- |
|  | **MACCE** | | **All-cause death** | | **MI** | | **Stroke** | | **URR** | |
| **Variables** | P value | HR (95% CI) | P value | HR (95% CI) | P value | HR (95% CI) | P value | HR (95% CI) | P value | HR (95% CI) |
| Control | - | 1.00 (Reference) | - | 1.00 (Reference) | - | 1.00 (Reference) | - | 1.00 (Reference) | - | 1.00 (Reference) |
| DM alone | **0.012** | 1.31 (1.06-1.61) | **0.019** | 1.57 (1.08-2.28) | 0.9 | 0.94 (0.36-2.42) | **0.044** | 2.24 (1.02-4.93) | 0.4 | 1.12 (0.87-1.44) |
| Obese alone | 0.15 | 1.13 (0.96-1.32) | 0.11 | 0.73 (0.50-1.08) | 0.7 | 1.13 (0.61-2.11) | 0.5 | 1.25 (0.62-2.53) | 0.13 | 1.15 (0.96-1.37) |
| Obese and DM | 0.12 | 1.16 (0.96-0.12) | 0.5 | 1.16 (0.79-1.70) | 0.067 | 0.36 (0.12-1.07) | 0.4 | 1.37 (0.64-2.94) | 0.2 | 1.16 (0.94-1.43) |
| Age | 0.8 | 1.00 (0.99-1.01) | **<0.001** | 1.06 (1.05-1.08) | 0.07 | 0.98 (0.95-1.00) | **0.043** | 1.03 (1.00-1.06) | **0.004** | 0.99 (0.98-1) |
| Sex | >0.9 | 1.01 (0.85-1.18) | >0.9 | 1.02 (0.73-1.41) | 0.2 | 1.77 (0.74-4.23) | 0.6 | 1.18 (0.64-2.19) | 0.7 | 0.97 (0.80-1.17) |
| Smoking | 0.8 | 0.99 (0.80-1.08) | 0.50 | 1.08 (0.89-1.31) | 0.2 | 1.24 (0.89-1.74) | 0.095 | 0.71 (0.48-1.06) | 0.8 | 1.01 (0.92-1.11) |
| Hypertension | **0.001** | 1.25 (1.09-1.43) | 0.6 | 1.08 (0.80-1.45) | 0.2 | 1.46 (0.83-2.58) | 0.084 | 1.66 (0.93-2.95) | **0.003** | 1.25 (1.08-1.46) |
| Previous MI | 0.073 | 1.23 (0.98-1.53) | **0.020** | 1.61 (1.08-2.42) | 0.7 | 1.20 (0.50-2.84) | 0.081 | 2.00 (0.92-4.36) | 0.2 | 1.17 (0.91-1.52) |
| Peripheral vascular disease | 0.5 | 1.10 (0.84-1.46) | **0.028** | 1.68 (1.06-2.68) | 0.4 | 0.41 (0.05-3.00) | 0.2 | 0.24 (0.03-1.79) | 0.8 | 0.96 (0.68-1.35) |
| Cerebrovascular disease | 0.7 | 0.97 (0.80-1.18) | 0.6 | 0.89 (0.62-1.30) | 0.9 | 1.07 (0.45-2.57) | **<0.001** | 2.80 (1.55-5.07) | 0.5 | 0.92 (0.73-1.16) |
| Renal insufficiency | **0.009** | 1.34 (1.07-1.67) | **<0.001** | 2.68 (1.93-3.73) | 0.8 | 0.87 (0.26-2.86) | 0.9 | 1.09 (0.46-2.59) | 0.6 | 0.92 (0.68-1.24) |
| Multi-vessel coronary disease | **0.003** | 1.27 (1.09-1.49) | **0.034** | 1.52 (1.03-2.25) | 0.14 | 1.73 (0.83-3.59) | 0.061 | 0.57 (0.32-1.03) | **0.008** | 1.26 (1.06-1.51) |
| Albumin | **0.047** | 0.98 (0.97-1.00) | **0.009** | 0.95 (0.92-0.99) | **<0.001** | 0.86 (0.81-0.92) | 0.3 | 0.97 (0.90-1.04) | 0.6 | 0.99 (0.97-1.01) |
| Cardiac ejection fraction | 0.2 | 1.01 (1.00-1.01) | **0.003** | 0.98 (0.96-0.99) | 0.8 | 1.00 (0.97-1.04) | 0.2 | 0.98 (0.95-1.01) | **0.007** | 1.01 (1.00-1.02) |
| Primary PCI | 0.4 | 1.07 (0.91-1.26) | 0.3 | 1.22 (0.86-1.73) | 0.8 | 0.92 (0.48-1.77) | 0.5 | 1.27 (0.67-2.40) | 0.6 | 1.04 (0.87-1.25) |
| MACCE, major adverse cardiac and cerebrovascular events; MI, myocardial infarction; URR, unplanned repeat revascularization; DM, diabetes mellitus; CI, confidence interval;  HR, hazard ratio; PCI percutaneous coronary intervention; Bold indicates *P* < 0.05. | | | | | | | | | | |

| Supplementary Table 4. Sensitivity analysis for deleting heart failure patients | | | | | | | | | |  |
| --- | --- | --- | --- | --- | --- | --- | --- | --- | --- | --- |
| **Outcomes** | **DM**  **Category** | **BMI**  **Category** | **Event Rate**  n (%) | **Unadjusted Model** | | | **Adjusted Model** | | | ***P-***interaction |
|  |  |  |  | HR | 95% CI | ***P***  value | HR | 95% CI | ***P***  value |  |
| MACCE | Non-DM | Normal-weight | 249（44.1%） | 1(Reference) | | | | | | 0.439 |
|  |  | Overweight and Obesity | 341（48.9%） | 1.17 | 0.99, 1.38 | 0.059 | 1.13 | 0.96, 1.34 | 0.15 |  |
|  | DM | Normal-weight | 113（52.0%） | 1(Reference) | | | | | |  |
|  |  | Overweight and Obesity | 183（54.1%） | 0.97 | 0.77, 1.23 | 0.8 | 1.05 | 0.82, 1.35 | 0.7 |  |
| All-cause death | Non-DM | Normal-weight | 52（9.2%） | 1(Reference) | | | | | | 0.485 |
|  |  | Overweight and Obesity | 41（5.8%） | 0.67 | 0.44, 1.01 | 0.053 | 0.77 | 0.50, 1.18 | 0.2 |  |
|  | DM | Normal-weight | 30（13.8%） | 1(Reference) | | | | | |  |
|  |  | Overweight and Obesity | 40（11.8%） | 0.79 | 0.49, 1.27 | 0.3 | 1.01 | 0.61, 1.67 | >0.9 |  |
| MI | Non-DM | Normal-weight | 17（3.0%） | 1(Reference) | | | | | | 0.109 |
|  |  | Overweight and Obesity | 26（3.7%） | 1.23 | 0.67, 2.27 | 0.5 | 1.22 | 0.65, 2.30 | 0.5 |  |
|  | DM | Normal-weight | 5（2.3%） | 1(Reference) | | | | | |  |
|  |  | Overweight and Obesity | 3（0.8%） | 0.37 | 0.09, 1.53 | 0.2 | 0.34 | 0.08, 1.47 | 0.15 |  |
| Stroke | Non-DM | Normal-weight | 11（1.9%） | 1(Reference) | | | | | | 0.226 |
|  |  | Overweight and Obesity | 17（2.4%） | 1.23 | 0.58, 2.62 | 0.6 | 1.31 | 0.60, 2.89 | 0.5 |  |
|  | DM | Normal-weight | 11（5.0%） | 1(Reference) | | | | | |  |
|  |  | Overweight and Obesity | 13（3.8%） | 0.69 | 0.31, 1.54 | 0.4 | 0.63 | 0.27, 1.45 | 0.3 |  |
| URR | Non-DM | Normal-weight | 205（36.3%） | 1(Reference) | | | | | | 0.717 |
|  |  | Overweight and Obesity | 299（42.9%） | 1.22 | 1.02, 1.46 | **0.026** | 1.14 | 0.95, 1.37 | 0.2 |  |
|  | DM | Normal-weight | 82（37.7%） | 1(Reference) | | | | | |  |
|  |  | Overweight and Obesity | 146（43.1%） | 1.09 | 0.83, 1.43 | 0.7 | 1.11 | 0.84, 1.48 | 0.5 |  |
| Covariates for the adjusted model: age, sex, smoking, previous myocardial infarction, hypertension, cerebrovascular disease, peripheral vascular disease, renal insufficiency, albumin, cardiac ejection fraction, multi-vessel coronary disease and primary percutaneous coronary intervention; BMI, body mass index; DM, diabetes mellitus; CI, confidence interval; HR, hazard ratio; MACCE, major adverse cardiac and cerebrovascular events; MI, myocardial infarction; URR, unplanned repeat revascularization; Bold indicates *P* < 0.05. | | | | | | | | | | |
